# Enhanced osteogenic potential of chitosan scaffolds loaded with *Lawsonia inermis*, hydroxyapatite particles, and dental pulp stem cells in rats

**DOI:** 10.1016/j.reth.2026.101171

**Published:** 2026-09-16

**Authors:** Maryam Karandish, Golnaz Ziaei, Fatemeh Akbarizadeh, Negar Azarpira, Shahrokh Zare, Ahmad Vaez, Nader Tanideh

**Affiliations:** a Orthodontic Department, Dental School, Shiraz University of Medical Sciences, Shiraz, Iran; b Orthodontic Research Center, School of Dentistry, Shiraz University of Medical Sciences, Shiraz, Iran; c Transplant Research Center, Shiraz University of Medical Sciences, Shiraz, Iran; d Stem Cells Technology Research Center, Shiraz University of Medical Sciences, Shiraz, Iran; e Department of Tissue Engineering and Applied Cell Sciences, School of Advanced Medical Sciences and Technologies, Shiraz University of Medical Sciences, Shiraz, Iran; f Histomorphometry and Stereology Research Center, Shiraz University of Medical Sciences, Shiraz, Iran; g Department of Pharmacology, Medical School, Shiraz University of Medical Sciences, Shiraz, Iran

**Keywords:** Scaffold, Chitosan, Bone regeneration, *Lawsonia inermis*, Hydroxyapatite particles, Dental pulp stem cells

## Abstract

**Introduction:**

Maxillary/alveolar bone reconstruction is vital for cleft and pre-prosthetic patients. Autogenous bone (iliac crest) is the gold standard for clefts due to its bone-forming ability. For large defects (trauma/tumor), the vascularized fibula flap is preferred for its strength and blood supply.

**Methods:**

This study investigates the physicochemical and biological properties of chitosan (Cs)-based scaffolds. Three scaffold formulations were prepared for in vitro characterization: pure Cs, Cs enriched with 8% *Lawsonia inermis* extract (Cs-LI), and Cs combined with LI and nanohydroxyapatite (Cs-LI-HAPs). For the in vivo study, five experimental groups were evaluated in a rat mandibular defect model: sham (empty defect), control (Cs scaffold alone), Cs-LI, Cs-LI-HAPs, and Cs-LI-HAPs seeded with 1 × 10^6^ dental pulp stem cells (DPSCs).

**Results:**

The synthesized HAPs showed a biomimetic apatite structure confirmed by FTIR and XRD, with a particle size of 730.2 ± 38.45 nm. Scaffold characterization demonstrated that Cs-LI-HAP had superior structural integrity, featuring interconnected porosity, improved mechanical strength (17.57 ± 1.49 MPa compressive strength), and a lower degradation rate (65.48 ± 10.35% at 4 weeks) compared to Cs and Cs-LI scaffolds. In vitro tests showed that Cs-LI-HAPs had good hemocompatibility and supported DPSC growth. In vivo, CBCT and histopathological analyses revealed that the Cs-LI-HAPs-DPSCs group exhibited significantly less fibrous tissue formation and greater bone regeneration compared to other groups.

**Conclusions:**

The Cs-LI-HAPs scaffold showed strong bone regeneration potential, with HAPs improving strength and stability, LI enhancing cell support, and DPSCs promoting new bone formation, making it a promising platform for maxillofacial defect repair.

## 1 Introduction

The reconstruction of maxillary and alveolar bone defects is crucial for craniofacial rehabilitation, especially for patients with cleft lip and palate or those needing pre-prosthetic orthodontics. Traditional bone grafting techniques are essential for restoring jaw continuity and function. The choice of graft material greatly affects treatment stability and success [1]. Autogenous bone grafts, taken from the patient's own body, are the gold standard because of their ability to promote bone growth and ensure good graft integration and long-term support for tooth movement. For alveolar clefts, the iliac crest is the preferred donor site due to its plentiful cancellous bone and good volume [2]. However, for larger maxillary defects caused by trauma, tumor removal, or congenital syndromes, the vascularized fibula flap often becomes the better option. Its strength and reliable blood supply support more complex and larger reconstructions [3].

New engineering methods for better bone regeneration have come about because of progress in biomaterials and tissue engineering. Bone tissue engineering has adopted an interdisciplinary approach in the pursuit of innovative and improved solutions to the biomedical engineering issues associated with bone regeneration [4]. Among the various scaffold fabrication techniques, freeze-dried sponge-type scaffolds have gained attention due to their interconnected porous architecture, which facilitates cell migration, nutrient exchange, and vascular ingrowth [5]. Chitosan (CS) is a biocompatible and osteoconductive polysaccharide. It has shown great promise as a scaffold material due to its structural similarity to glycosaminoglycans and its ability to improve cell-matrix interactions [6,7]. To enhance scaffold performance, bioactive compounds like *Lawsonia inermis* (LI) can be added. LI is known for its antimicrobial, antioxidant, and pro-angiogenic properties, which help reduce infection risks and improve tissue repair [8,9]. Additionally, hydroxyapatite particles (HAPs) have a mineral composition similar to bone and osteogenic potential. It can strengthen the scaffold structure while promoting osteoblast activity [4,10,11]. Human dental pulp stem cells (DPSCs) further support bone regeneration by turning into osteoblasts through endochondral or intramembranous ossification, depending on the environment of the defect [[12], [13], [14]].

To overcome the limitations of conventional bone grafts, tissue engineering strategies have focused on developing biomimetic scaffolds that combine biocompatible materials, bioactive agents, and stem cells. In this context, the present study reports the fabrication and characterization of chitosan-based scaffolds incorporating LI extract and HAPs, and evaluates their in vitro cytocompatibility with DPSCs and in vivo bone regenerative capacity in a rat mandibular defect model. The combination of chitosan's structural versatility, LI's anti-inflammatory and pro-angiogenic properties, HAP's osteoconductivity, and DPSCs' osteogenic potential is hypothesized to provide a conducive microenvironment for bone healing. The mandibular critical-size defect model was selected for this study because the mandible is a weight-bearing craniofacial bone with distinct anatomical and mechanical properties, making it a clinically relevant and challenging site for evaluating bone regeneration strategies. Furthermore, this model is well-established in preclinical bone tissue engineering research and allows for standardized assessment of scaffold performance under load-bearing conditions, which is directly translatable to maxillofacial and alveolar bone defects. Post-implantation assessments included cone-beam computed tomography (CBCT) and histopathological analyses to quantify new bone formation and scaffold integration over a two-month period.

## 2 Materials and methods

### 2.1 Materials

All chemicals and reagents used in this study were obtained from: Dulbecco's Modified Eagle's Medium/Nutrient Mixture F-12 (DMEM/F12), fetal bovine serum (FBS), collagenase type I, and trypsin-EDTA (Gibco), non-essential amino acids and dimethyl sulfoxide (DMSO), calcium oxide (CaO), phosphoric acid (H_3_PO_4_), ammonium hydroxide (NH_4_OH), and glutaraldehyde (25%), (Sigma-Aldrich), MTT assay kit (DNAbiotech, Iran), medium molecular weight chitosan (Iran Chitosan, Iran), adult male Sprague-Dawley rats (Pasteur Institute, Iran), ketamine (Bioveta) and xylazine (Kela).

### 2.2 Synthesis of HAPs

In this study, HAPs was prepared using CaO, H_3_PO_4_, and NH_4_OH as starting materials. First, 1.42 mol (79.55 g) of CaO was mixed with 500 mL of deionized water. This mixture was stirred continuously at 1000 rpm and 37°C for 24 h to create a Ca(OH)_2_ suspension. This step kept the mixture safe from contamination. Next, 97.32 g of 85% H_3_PO_4_ was added slowly at a rate of 1.5 mL per minute, while monitoring the pH to keep the reaction conditions exact. The mixture was stirred again at 1000 rpm and 20°C for 24 h to help it mature. To stabilize the supersaturated solution and achieve the target Ca/P ratio of 1.67, 0.28 mol (9.94 g) of NH_4_OH was introduced, raising the pH above 10 and ensuring single-phase HA formation. Following ripening, aliquots of the HA slurry were oven-dried (100°C, 1 h) and sintered at 700°C for 1 h. The cooled samples were pulverized via ball milling for subsequent analysis [15,16].

### 2.3 Characterization of HAPs

#### 2.3.1 Morphology and size evaluations

The surface structure of HAPs was examined with a Philips XL-30 scanning electron microscope (SEM). Before imaging, the samples were coated with a thin layer of gold using an EMITECH K450X sputter coater from England. The particle size distribution of HAPs was also assessed through dynamic light scattering (DLS) analysis.

#### 2.3.2 Chemical composition and structural analysis

The functional groups of HAPs were examined using FTIR spectroscopy (Thermo Nicolet NEXUS 670). We prepared samples as KBr pellets and collected spectral data within the wavenumber range of 500 to 4500 cm^−1^, with a resolution of 4 cm^−1^ and a scanning speed of 120 mV/min [17]. In addition, we conducted XRD analysis on a Bruker Advance D8 diffractometer to assess the crystalline structure and phase composition of the synthesized materials. To reduce background noise, we placed powdered samples on a silicon zero-background holder during measurements. This technique helps clarify the crystalline properties, phase changes in HAPs, and their interactions with the surrounding matrix [18].

### 2.4 Preparation of LI extract

Hydroalcoholic extraction of LI used a standardized maceration method. Two hundred grams of finely powdered henna leaves soaked in 1000 mL of 70% ethanol for 72 h at room temperature (25 ± 2°C) with periodic mixing. We filtered the resulting extract through Whatman No. 1 filter paper with a Büchner funnel under vacuum to remove solid particles. To keep the phytochemicals stable, we split the concentrated extract into amber glass vials lined with aluminum foil to stop photodegradation. We then stored the vials at 4°C in a temperature-controlled refrigerator [19].

### 2.5 Scaffolds fabrication

The freeze-drying method was used for fabricating Cs scaffolds. The process involves dissolving Cs in acetic acid (2% v/v). The solution was homogenized and frozen at −80°C to control pore formation. The frozen samples are then lyophilized, which removes ice through sublimation to create a 3D porous scaffold. To determine the optimal concentration of LI extract for scaffold fabrication, different concentrations of LI (4%, 8%, 16%, and 32%) were incorporated into Cs scaffolds. Based on the MTT assay results, 8% LI was selected as the optimal concentration for further studies. Similarly, to optimize the HAPs content, different concentrations of HAPs (4%, 8%, 16%, and 32%) were incorporated into Cs scaffolds. Since there was no significant difference in cell viability among the HAPs concentrations (based on MTT assay results), the 8% HAPs concentration was selected to ensure adequate porosity and mechanical integrity while maintaining favorable biological properties [20,21]. Accordingly, scaffolds were prepared as Cs, Cs-LI (containing 8% LI), and Cs-LI-HAPs (containing 8% LI and 8% HAPs). To improve mechanical stability, scaffolds were cross-linked with 25% glutaraldehyde vapor. This was followed by three thorough washing steps with deionized water (30 min each) to remove toxic residues. After washing, the scaffolds were subjected to a second lyophilization step to ensure complete removal of residual solvents and moisture. Finally, sterilization was performed using UV irradiation for 30 min on each side [22].

### 2.6 Characterization of scaffolds

#### 2.6.1 Morphology assessment

The morphological and microstructural features of the fabricated scaffolds were examined using SEM to assess pore architecture, surface topography, and interconnectivity. Prior to imaging, the samples were subjected to sputter-coating.

#### 2.6.2 Weight loss study

The in vitro degradation profiles of the scaffolds were systematically investigated by incubating specimens in phosphate-buffered saline (PBS, pH 7.4) maintained at 37°C under static conditions to simulate physiological environments [23]. At predetermined time intervals (7, 14, and 21 days), triplicate samples were extracted from PBS, gently rinsed with deionized water, and lyophilized to determine residual dry mass. The percentage weight loss, serving as a quantitative indicator of degradation, was calculated using Equation (1) (Eq. (1)):

(Eq.1) $$Weight\;loss\;\left(\%\right)=\frac{W_{0}-W_{1}}{W_{0}}\times 100$$

where *W*_0_​ is the initial weight of the hydrogels and scaffolds, and *W*_1_​ is the dry weight after removal from PBS.

#### 2.6.3 Swelling study

The swelling behavior of the scaffolds was studied by placing pre-weighed dry samples (n = 3 per experimental group) in PBS (pH 7.4) at room temperature [22]. At set time points, samples were taken out of the PBS, and extra surface liquid was removed by blotting with pre-weighed filter paper (Whatman Grade 1) for exactly 10 s. This ensured uniform fluid removal for all specimens. The swelling ratio, expressed as a percentage, was calculated using Eq. (2):

(Eq.2) $$Equilibrium\;mass\;swelling\;\left(\%\right)=\frac{W_{t}-W_{0}}{W_{0}}\times 100$$

where *W*_*t*_ denotes the swollen mass at measurement time point t, and *W*_0_​ represents the initial dry mass of the scaffolds prior to immersion. Measurements continued until the hydrogels reached equilibrium.

#### 2.6.4 Porosity evaluation

The architectural porosity of the fabricated scaffolds was quantitatively evaluated using an ethanol-based liquid displacement technique [24]. Precisely weighed lyophilized samples (n = 5 per group) were immersed in a graduated glass cylinder containing a known volume of absolute ethanol (V_1_). The samples were subjected to ethanol. The total volume (V_2_) was recorded after visible air bubble cessation. Subsequently, samples were carefully removed, allowing 30 s for excess surface ethanol to drain before measuring the residual ethanol volume (V_3_). The percentage porosity was calculated according to Eq. (3):

(Eq.3) $$Porosity\;\left(\%\right)=\frac{V_{1}-{\;V}_{3}}{V_{2}-V_{3}}\times 100$$

#### 2.6.5 Mechanical properties test

The compressive strength of 1 × 1 × 1 cm^3^ scaffolds was measured following ASTM F2150-02 standards using a uniaxial tensile-compression testing machine (SANTAM STM 20, Iran) at a rate of 1 mm/min. Five samples from each group were tested, and average values were computed. Key mechanical properties were obtained from stress-strain curves, where stress (σ) and strain (*ε*) were calculated using Eqs. (5) and (6):

(Eq.5) $$Stress\;\left(\sigma \right)=\frac{F}{A_{0}}$$

where F is force, and A_0_ is the original cross-sectional area of the scaffold.

(Eq.6) $$Strain\;\left(\varepsilon \right)=\frac{\Delta L}{L_{0}}$$

where ΔLi s Extension, and $L_{0}$ is original gauge length of the scaffold.

The elastic modulus (E) was determined from the linear elastic region of the curve using Eq. (7):

(Eq.7) $$Elastic\;Modulus\;\left(E\right)=\frac{\Delta \sigma }{\Delta \varepsilon }$$

where Δσ and Δε, in the linear region of the stress-strain curve, are changes in stress and strain, respectively.

Yield strength was identified as the stress where plastic deformation begins, using the 0.2% offset method. The ultimate compression strength (UCS) represented the maximum stress before failure, while elongation at break was recorded as the percent increase in length at failure [25].

#### 2.6.6 Contact angle test

This study looked at how well tissue engineering scaffolds repel or attract water using the sessile drop method. This technique measures the contact angle of a water droplet on the scaffold surface. A lower contact angle (<90°) shows higher hydrophilicity, which helps improve cellular interactions and biocompatibility. Using a precise contact angle analyzer, we placed 10 μL droplets of deionized water on lyophilized scaffold samples. We took high-resolution images to calculate the average contact angle. The experiment was conducted with five repeats for each group, ensuring reliable and repeatable results [26].

#### 2.6.7 Hemocompatibility test

The hemocompatibility of tissue-engineered scaffolds was assessed using a standardized hemolysis assay. This test is crucial for understanding how blood interacts with materials, which affects blood clotting and inflammation. Whole blood (2 mL) from fresh Sprague Dawley rats was collected in sodium citrate anticoagulant (3.8% w/v, 250 μL) and then diluted with phosphate-buffered saline (PBS, 2.5 mL). The test materials were incubated with the diluted blood for 60 min at 37°C. After centrifugation, we measured hemoglobin release using a spectrophotometer at 545 nm. We included strict controls, such as a positive control to induce 100% hemolysis with deionized water and a negative control to show 0% hemolysis in PBS. The percentage hemolysis was calculated using Eq. (8):

(Eq.8) $$Hemolysis\;\left(\%\right)=\frac{D_{t}-D_{nc}}{D_{pc}-D_{nc}}\times 100$$

D_t_, D_nc_, and D_pc_ are the absorbances of the samples, NC and PC, respectively [22].

#### 2.6.8 Human DPSCs isolation

The use of human dental pulp tissue was approved by the ethical committee of Shiraz University of Medical Sciences under approval code IR.SUMS.AEC.1403.051. All experimental protocols involving human tissue were performed in accordance with the relevant guidelines and regulations. Informed consent was also obtained from all subjects. DPSCs were isolated from healthy extracted teeth obtained from two consenting patients under sterile conditions (informed consent was obtained from subjects). Immediately following extraction, pulp tissue was collected and washed three times with PBS before mechanical mincing into 1-2 mm^3^ fragments. Enzymatic digestion was performed in 0.14% collagenase type I for 45 min at 37°C (5% CO_2_), followed by neutralization with DMEM/F-12 containing 10% FBS. After centrifugation (200 ×g, 7 min), the cellular pellet was resuspended in complete culture medium. This medium consisted of DMEM/F-12, which was supplemented with 10% FBS, 1% penicillin-streptomycin, and 1% non-essential amino acids. The cells were kept at 37°C in a humidified 5% CO_2_ atmosphere. Once the cells reached 70% confluence, they were passaged using 0.25% trypsin-EDTA. The cells were expanded through three passages to get enough cell numbers for the next experiments [27].

#### 2.6.9 Cell viability assay

The cytocompatibility of scaffolds was evaluated using the MTT viability assay with human DPSCs isolated as described in Section 2.6.8. The cells were grown in DMEM/F12 with 10% FBS, 100 U/mL penicillin, and 100 μg/mL streptomycin under standard culture conditions at 37°C, 5% CO_2_, and 95% humidity. To assess cytotoxicity, cells were seeded onto sterile scaffolds in 96-well plates at a density of 1 × 10^4^ cells per well. At predetermined time points (days 1, 3, and 5), culture media were replaced with 200 μL of MTT solution, followed by 4 h of incubation under dark conditions at 37°C to allow formazan crystal formation. The supernatant was carefully aspirated, and insoluble formazan crystals were solubilized with 100 μL DMSO. Metabolic activity was quantified by measuring absorbance at 570 nm using a microplate reader, with values directly correlating to viable cell numbers [22].

#### 2.6.10 Cell migration test

To assess the biological potential of the fabricated scaffolds and lyophilized hydrogels, extract solutions were prepared following ISO 10993-5 cytocompatibility testing guidelines. Sterilized scaffold samples (3 cm^2^ surface area) were immersed in DMEM and incubated in a shaking incubator at 37°C for two weeks in the following groups: culture medium (Sham), Cs (Control), Cs-LI, and Cs-LI-HAPs. After incubation, each extract was sterile-filtered through a 0.22 μm membrane filter. The resulting extracts were then supplemented with 5% fetal bovine serum (FBS) and a penicillin/streptomycin antibiotic cocktail (100 units/ml and 100 μg/ml, respectively) [22].

To assess the gap closure potential of the extracts, a cell migration assay was conducted using confluent MC3T3 osteoblast precursor monolayers in 24-well plates. A standardized cell gap was created by scraping with a 200 μl pipette tip, followed by PBS washes to remove debris. The cells were then treated with 500 μl of scaffold extracts. Over 72 h of incubation (37°C, 5% CO_2_), cell migration into the gap area was monitored (0 h and 72 h). Quantitative analysis of gap closure was performed using Eq. (9):

(Eq.9) $$Gap\;closure\;\left(\%\right)=1-\frac{At}{A0}\times 100$$

where A_t_ and A_0_ are the cell gap area at a specific time and the initial gap area, respectively [28].

### 2.7 In vivo study

All animal experiments were performed in accordance with the ARRIVE guidelines and in accordance with National Institutes of Health guide for the care and use of Laboratory animals (Eighth Edition, 2011), and the study protocol was also approved by the institutional ethics committee of Shiraz University of Medical Sciences (IR.SUMS.AEC.1403.051). This study used twenty-five 6-month-old male Sprague-Dawley rats (230 ± 20 g) to assess the bone-forming potential of scaffold formulations in large mandibular defects. The rats lived under normal lab conditions with free access to food and water. Under general anesthesia, which was induced by an intraperitoneal injection of ketamine (10 mg/kg) and xylazine (2 mg/kg), standardized critical-sized defects measuring 5 mm in diameter and 2 mm deep were surgically created in the mandibular bone, located behind the incisors, using a trephine burr. Animals were randomly allocated to five experimental groups (n = 5/group): sham (empty defect), control (Cs scaffold alone), Cs-Li, Cs-Li-HAPs, Cs-Li-HAPs seeded with 1 × 10^6^ DPSCs (Table 1). Pre-implantation, scaffolds for group 5 were pre-seeded with passage 3-5 DPSCs at a density of 1 × 10^6^ cells/scaffold. Following precise scaffold placement into defects, surgical sites were irrigated with normal saline and closed in layers using 3/0 silk sutures. Postoperative care included daily monitoring to ensure proper wound healing and animal welfare.

**Table 1:** Summary of experimental groups and their applications.

Group No.	Group Name	Description
1	Sham	Defect without treatment
2	Control	Cs scaffold alone
3	Cs-LI	Cs + 8% LI extract
4	Cs-LI-HAPs	Cs + 8% LI + 8% HAPs
5	Cs-LI-HAPs-DPSCs	Cs-LI-HAPs seeded with 1 × 10^6^ DPSCs

### 2.8 Statistical analysis

Data were analyzed using GraphPad Prism (v. 10) with one-way ANOVA followed by Tukey's post hoc test. Results are reported as mean ± SD, with statistical significance at p < 0.05.

## 3 Results and discussion

### 3.1 Characterization of HAPs

#### 3.1.1 Morphology and size analysis of HAPs

**Fig. 1:**
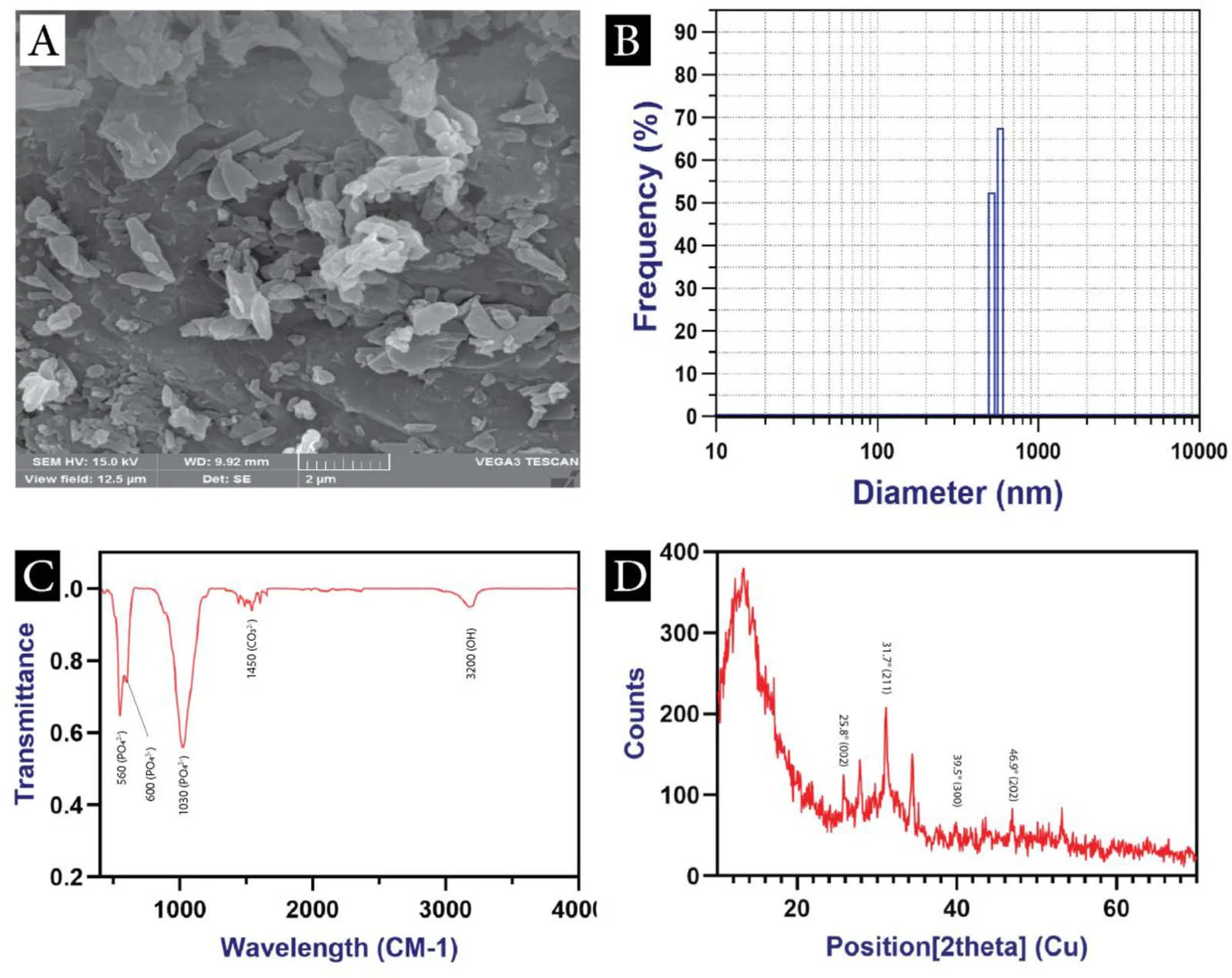
(A) Morphological analysis with SEM with size distribution, and (B) DLS analysis of HAPs, C) FTIR analysis of HAPs reveals OH^−^ stretching, CO_3_^2−^ substitution, and PO_4_^3−^ vibrational modes, consistent with biomimetic apatite, and D) XRD pattern of HAPs confirms its characteristic hydroxyapatite structure, with primary reflections matching standard HAPs peaks. Additional minor peaks suggest the presence of secondary phases or structural variations within the sample.

The structure of the synthesized HAPs was examined using SEM, as shown in Fig. 1A. A quantitative assessment of the SEM images, along with DLS analysis in Fig. 1B, showed that the HAPs particles had a uniform size distribution. The average particle diameter was 730.2 ± 38.45 nm. These methods provided a thorough evaluation of the structural features and size properties of the HAPs particles.

#### 3.1.2 Chemical composition analysis of HAPs

The FTIR spectrum shows absorption bands that are typical for HAPs. As shown in Fig. 1C, the stretching band around 3200 cm^−1^ comes from OH^−^ groups due to stretching vibrations. The peaks between 1400 and 1600 cm^−1^ indicate CO_3_^2−^ incorporation, which is common in biomimetic HAPs. The bands located at **∼**560, **∼**600, and **∼**1030 cm^−1^ originate from PO_4_^3−^ ions [29,30]. The XRD pattern of the HAPs (Fig. 1D) shows characteristic peaks at 2θ ∼ 25.8° (002), 31.7° (211), 39.5° (300), and 46.9° (202), which are typical for hydroxyapatite, confirming the material's identity as HAPs.

However, additional peaks at approximately 2θ = 27°, 34.5°, and 55.5° suggest the presence of other features or phases within the sample. The peak at 2θ around 27° might relate to structural distortions or satellite reflections connected to the main (002) reflection of HAPs because of its closeness. On the other hand, the peak at 2θ around 34.5° does not directly match standard HAPs reflections and may indicate a minor impurity phase, possibly tricalcium phosphate (TCP) or other overlapping reflections [31]. The peak at 2θ around 55.5° is noticeably higher than typical HAPs peaks and could be linked to an impurity phase such as carbonates or another crystalline component.

### 3.2 Characterization of scaffolds

#### 3.2.1 Morphology assessment

**Fig. 2:**
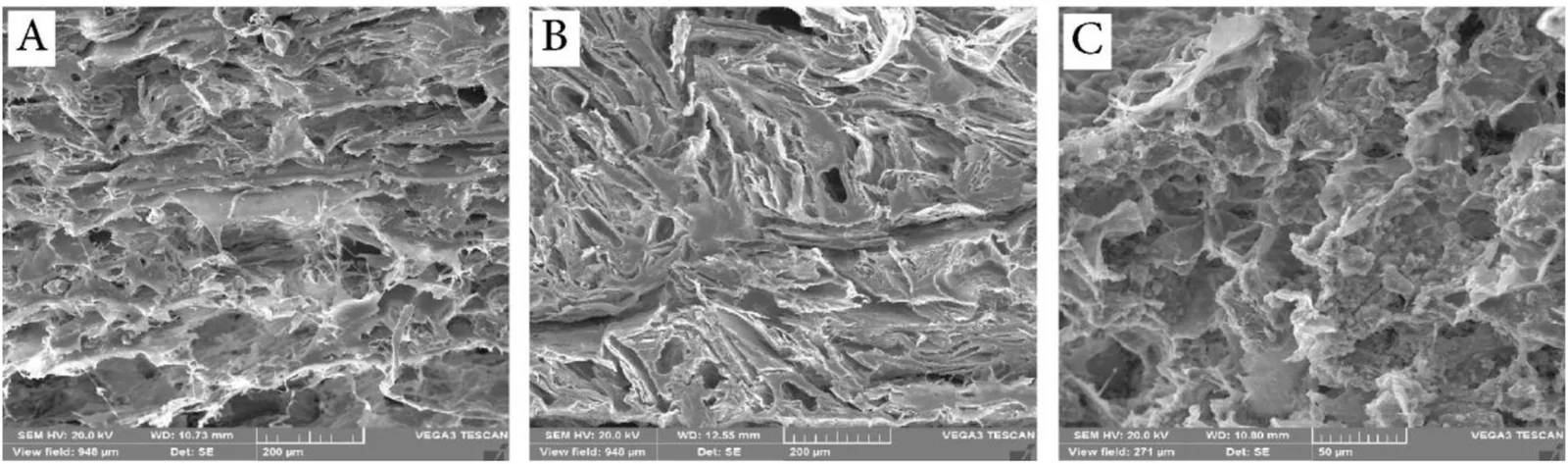
SEM images from Cs, Cs-LI, and Cs-LI-HAPs scaffolds. Cs-LI-HAPs composite scaffold displaying optimized porosity and particle distribution for improved performance.

SEM analysis revealed distinct morphological differences among the freeze-cast Cs (Fig. 2A), Cs-LI (Fig. 2B), and Cs-LI-HAPs scaffolds (Fig. 2C). The pure Cs scaffold displayed a fibrous texture with irregular pore distribution, while Cs-LI exhibited a layered architecture with narrow pores, potentially limiting cell infiltration. The Cs-LI-HAPs scaffold demonstrates the most advanced microstructural features. Its surface is highly rugged and granular, with well-defined, uniform pores that are larger and more interconnected than those in the Cs-LI but less than pure Cs scaffolds. The granular texture indicates the presence of HAPs, which is evenly dispersed throughout the matrix.

#### 3.2.2 Weight loss analysis

**Fig. 3:**
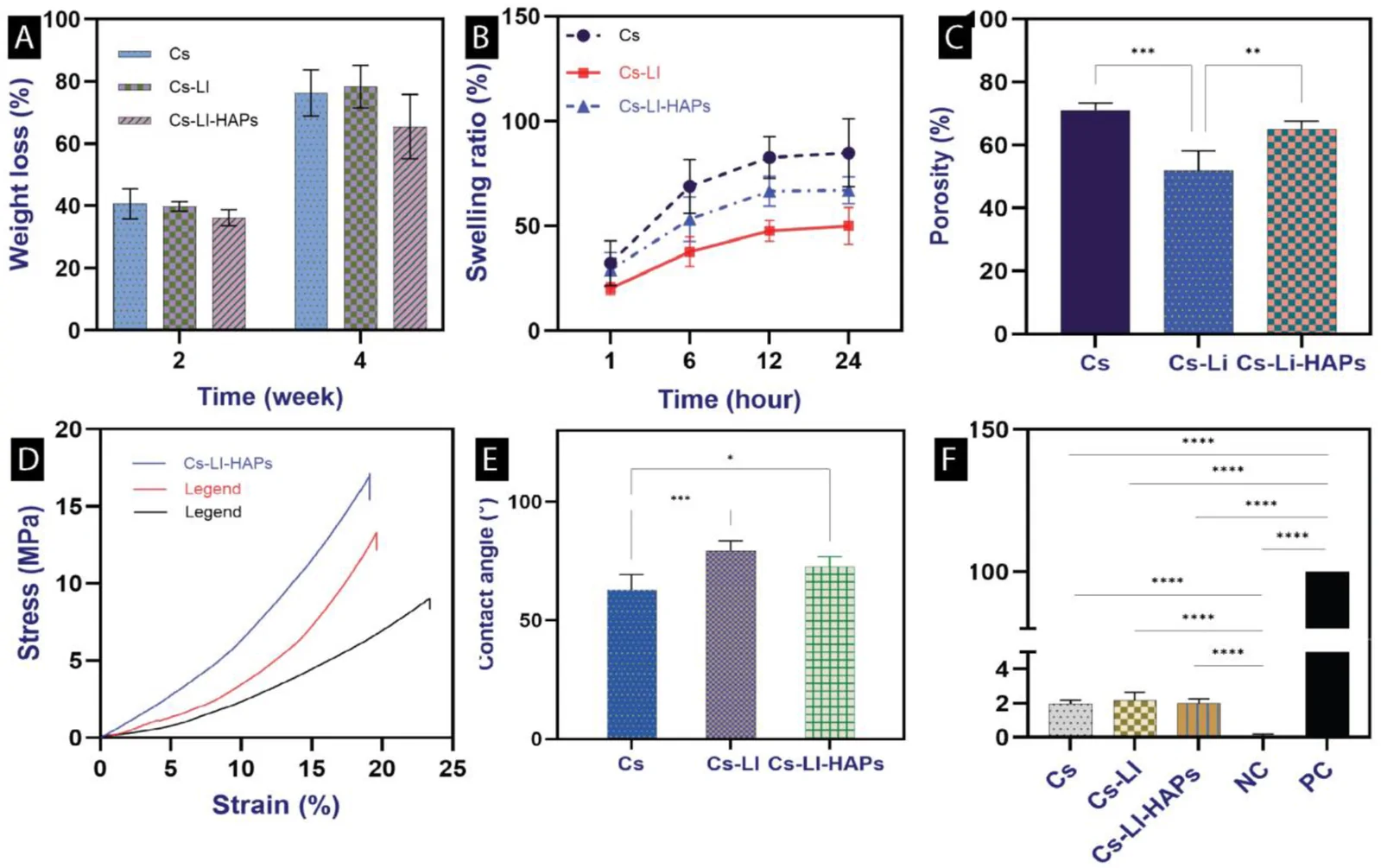
Degradation profiles of Cs, Cs-LI, and Cs-LI-HAPs scaffolds over 4 weeks **(**A), swelling ratio (B), porosity assessment (C), mechanical properties (D), contact angle measurement (E), and hemocompatibility evaluation (F) among experimental groups.

The Cs scaffolds exhibited a degradation percentage of 40.69 ± 4.80%, compared to 39.81 ± 1.56% for the Cs-LI and 36.18 ± 2.56% for the Cs-LI-HAPs scaffolds after 2 weeks. Also, the degradation percentages were 76.18 ± 7.42%, 78.31 ± 6.86%, and 65.48 ± 10.35% for Cs, Cs-LI, and Cs-LI-HAPs after 4 weeks (Fig. 3A). The observed decrease in weight loss may result from the influence of LI and HAPs on the polymer matrix, potentially modifying its crystallinity or degree of crosslinking. Such behavior is consistent with prior research reporting that the addition of particles to polymeric systems enhances mechanical performance and slows degradation [32,33].

#### 3.2.3 Swelling studies

The swelling capacity of scaffolds, a critical parameter influencing nutrient diffusion and mechanical stability during bone healing, was assessed through gravimetric analysis in PBS at 37°C. All scaffold formulations exhibited characteristic time-dependent swelling kinetics, achieving equilibrium within 2 h (Fig. 3B). Notably, the incorporation of LI and HAPs significantly reduced swelling ratios compared to pure Cs scaffolds (p < 0.05), suggesting enhanced structural stability through crosslinking interactions between the composite components.

#### 3.2.4 Porosity assessment

The porosity of scaffolds is crucial for effective bone regeneration. It allows cells to move in, nutrients to flow, and waste to be removed, all while keeping the structure intact [34]. Our results showed that Cs-LI composites had much lower porosity than other scaffolds (Fig. 3C). This finding aligns with their reduced swelling capability. Previous studies indicate that adding LI decreases pore size by increasing polymer crosslinking and raises hydrophobicity, which is evident from the higher water contact angles [35,36]. The controlled porosity in Cs-LI scaffolds appears to strike a good balance. It allows sufficient pore connections for cells to move and nutrients to flow while providing better mechanical strength to support bone-forming cells. This implies that these composites could be particularly effective for bone regeneration in load-bearing scenarios.

#### 3.2.5 Mechanical properties

The compression test results show how pure Cs, Cs-LI, and Cs-LI-HAPs scaffolds respond to stress and strain. In the elastic region, Cs-LI-HAPs has the highest stiffness, indicated by the steepest slope. Cs-LI follows, with Cs having the least stiffness. As strain increases, Cs-LI-HAPs outshines the others in strength and toughness, showing the highest unconfined compressive strength (UCS). Meanwhile, Cs shows limited strength and toughness. Adding LI improves stiffness and strength, while adding HAPs boosts these properties even more due to strong interfacial interactions and reinforcement (Fig. 3D and Table 2).

**Table 2:**
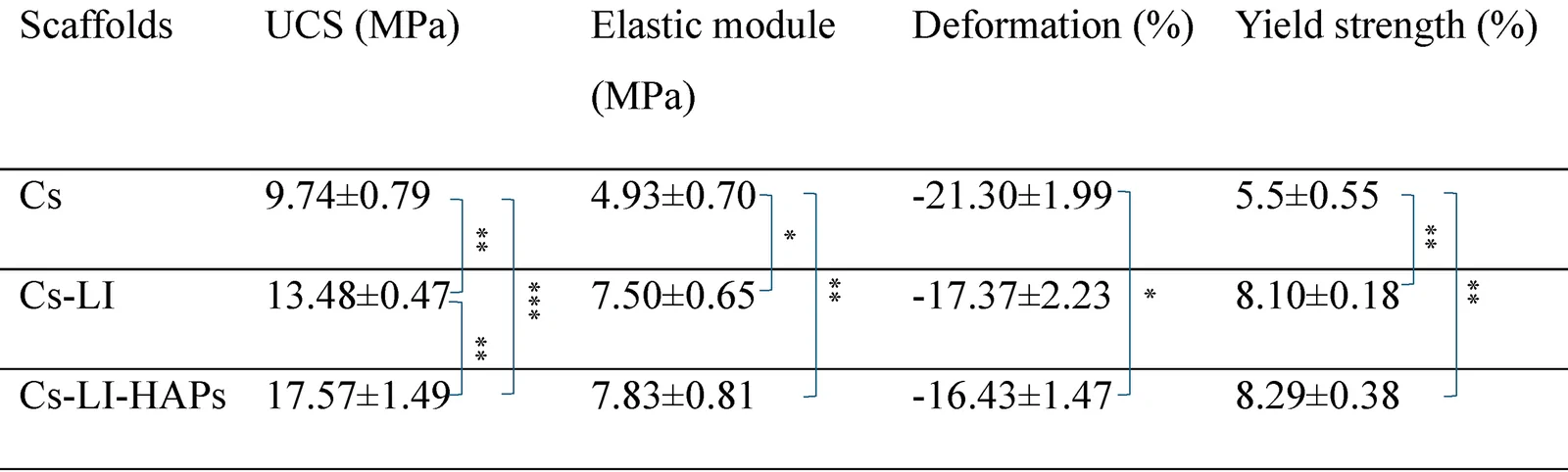
Mechanical properties of Cs, Cs-Li, and Cs-Li-HAPs scaffolds. n = 5; *p < 0.05, **p < 0.01, ***p < 0.001.

#### 3.2.6 Contact angle measurement

The wettability of a material is determined by its water contact angle, reflecting how effectively the surface can absorb cells, an essential factor for facilitating tissue regeneration [37]. Hydrophobicity is indicated by a high contact angle, while hydrophilicity is associated with a low angle. All tested scaffolds exhibited contact angles below 90°, falling within the optimal range for supporting cell attachment and growth. The contact angles of Cs, Cs-LI, and Cs-LI-HAPs scaffolds were 62.85 ± 6.465, 79.47 ± 4.018, and 72.62 ± 4.153 (Fig. 3E).

#### 3.2.7 Hemocompatibility test

The biocompatibility of biomaterials depends significantly on their interactions with blood constituents. Hemolysis, the rupture of red blood cells that causes hemoglobin release, is an important sign of blood compatibility. According to ASTM F756-08 guidelines, materials are categorized as non-hemolytic if they show less than 2% hemolysis, moderately hemolytic if they show between 2% and 5%, or hemolytic if they exceed 5% [38]. Experimental data revealed that both scaffolds and lyophilized hydrogels exhibited significantly lower hemolysis rates compared to the positive control (p < 0.001) [39,40]. All the tested scaffolds and hydrogels had hemolysis values within acceptable ranges, with hemocompatibility scores remaining below 5%. The hemocompatibility test results for the Cs, Cs-LI, and Cs-LI-HAPs scaffolds were 1.97 ± 0.20, 2.16 ± 0.49, and 1.99 ± 0.26 (Fig. 3F).

#### 3.2.8 Cell viability assay

**Fig. 4:**
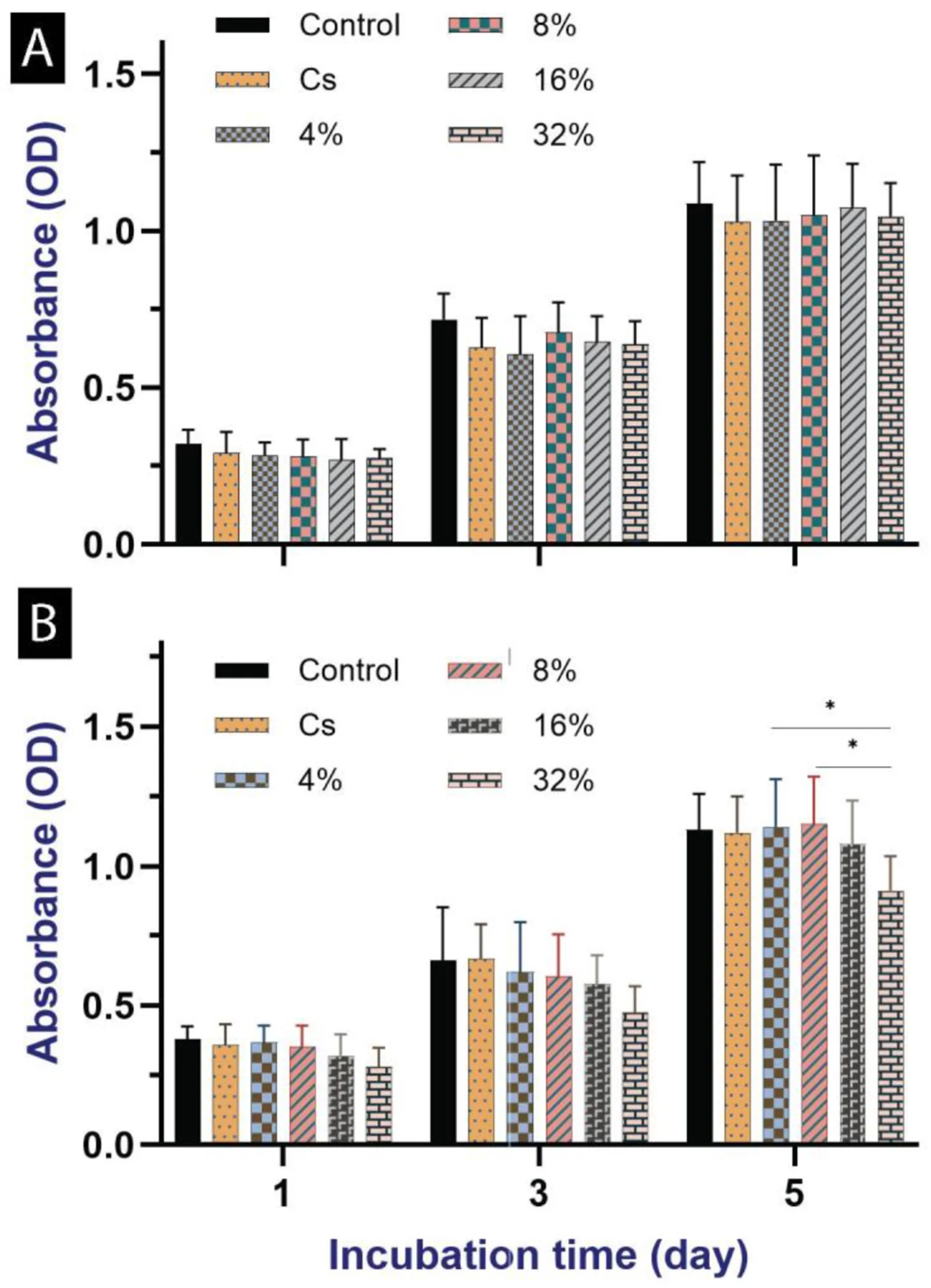
MTT assay demonstrates enhanced DPSCs proliferation on Cs scaffolds incorporating HAPs (A) and LI (B) compared to controls.

**Fig. 5:**
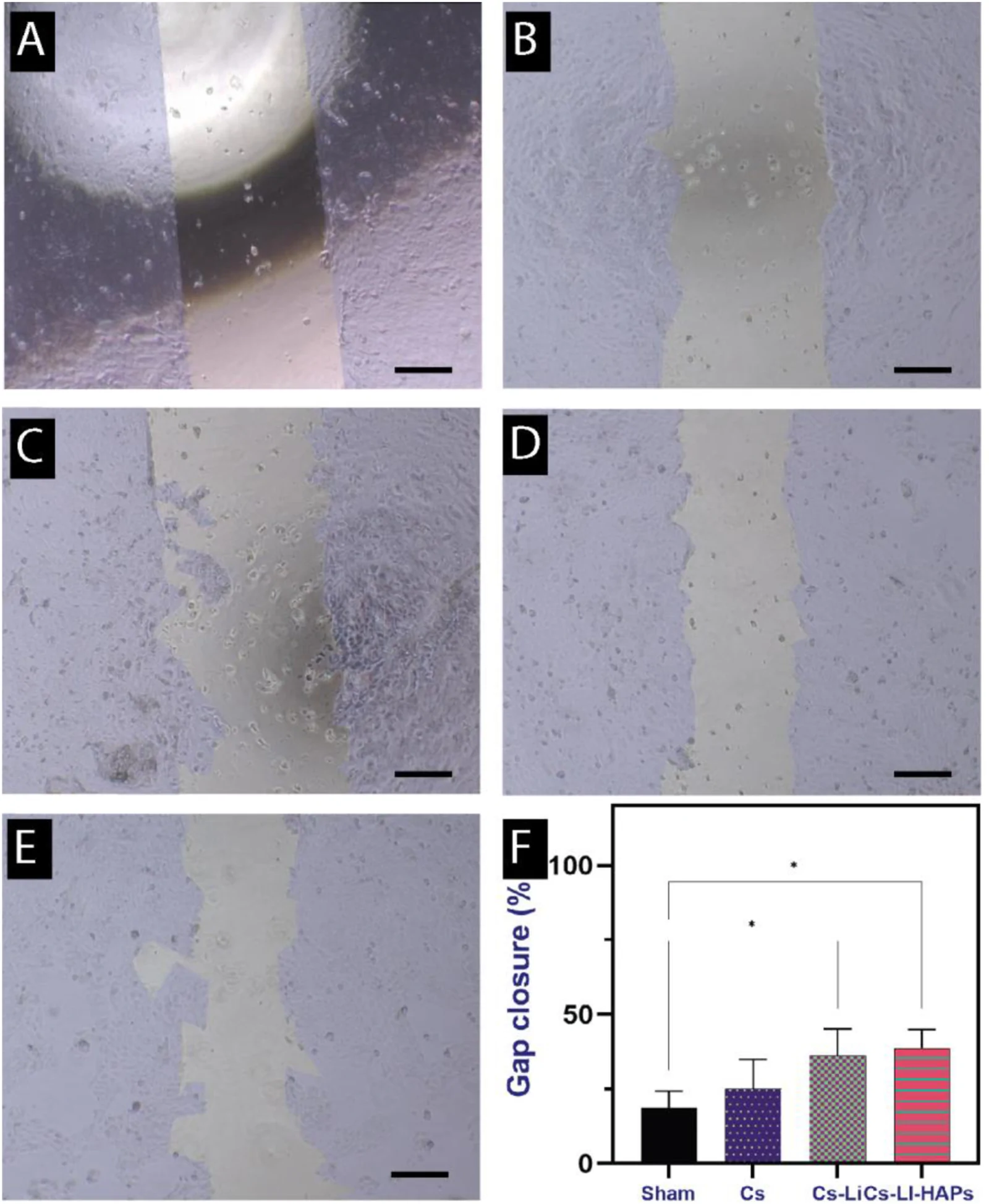
Enhanced keratinocyte migration by Cs-LI and Cs-LI-HAPs extracts. A cee gap was introduced in a confluent monolayer using a 200 μl pipette tip at the initial time point (A). Subsequently, cells were treated with either standard culture medium (Sham) (B), Cs (Control) (C), Cs-LI (D), and Cs-LI-HAPs (E) extracts for 72 h. Notably, groups supplemented with Cs-LI and Cs-LI-HAPs exhibited significantly enhanced migratory capacity relative to other extracts (F).

The MTT assay results in Fig. 4A and 5B assess how different concentrations of HAPs and LI affect the growth of DPSCs over 5 days. In Fig. 4A, adding HAPs to Cs scaffolds did not show any harmful effects. Likewise, Fig. 4B indicates that LI did not negatively impact DPSCs growth, with 4-8% LI showing the most improvement, but 16% and 32% did display toxic effects.

#### 3.2.9 Cell migration test

The impact of the samples’ extract on cell proliferation was evaluated using a cell migration assay conducted on MC3T3 cell monolayers. Notably, the groups treated with Cs-LI (36.28 ± 8.87%) and Cs-LI-HAPs (38.71 ± 6.20%) extracts showed significant migration rates vs sham (18.84 ± 5.40) and control (25.06 ± 9.91) (Fig. 5). The improved migratory response observed in these groups may be attributed to the combined action of bioactive components released from the materials.

It is also worth noting that despite the use of glutaraldehyde vapor for crosslinking, no signs of cytotoxicity, inflammatory reactions, or adverse tissue responses were observed in any of the experimental groups in MTT and migration tests. This confirms that the washing and sterilization protocol employed in this study was effective in removing residual glutaraldehyde and ensuring the biocompatibility of the scaffolds, consistent with previous reports [22].

### 3.3 In vivo study

#### 3.3.1 CBCT scan

**Fig. 6:**
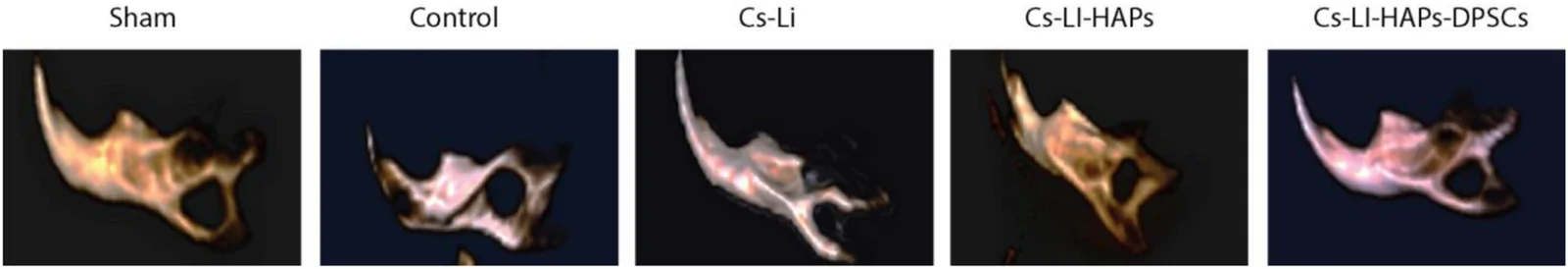
CBCT analysis demonstrates enhanced bone regeneration in defect sites treated with Cs containing LI, LI-HAPs, and LI-HAPs-DPSCs scaffolds compared to sham and control groups at 8 weeks post-surgery.

CBCT analysis at eight weeks post-surgery revealed no significant new bone formation in the defect site for the sham group. The areas of regenerated bone in the sham, control (Cs), Cs-LI, Cs-LI-HAPs, and Cs-LI-HAPs-DPSCs groups were 0.97 ± 0.17, 1.63 ± 0.27, 2.18 ± 0.39, 2.21 ± 0.45, and 2.75 ± 0.31 mm^2^, respectively. Notably, scaffolds containing LI demonstrated a measurable reduction in the residual defect area compared to the sham and control groups. The most significant improvement in bone regeneration was observed in the Cs-LI-HAPs-DPSCs group, indicating a combined effect of these bioactive components (Fig. 6).

#### 3.3.2 Histopathological findings

**Fig. 7:**
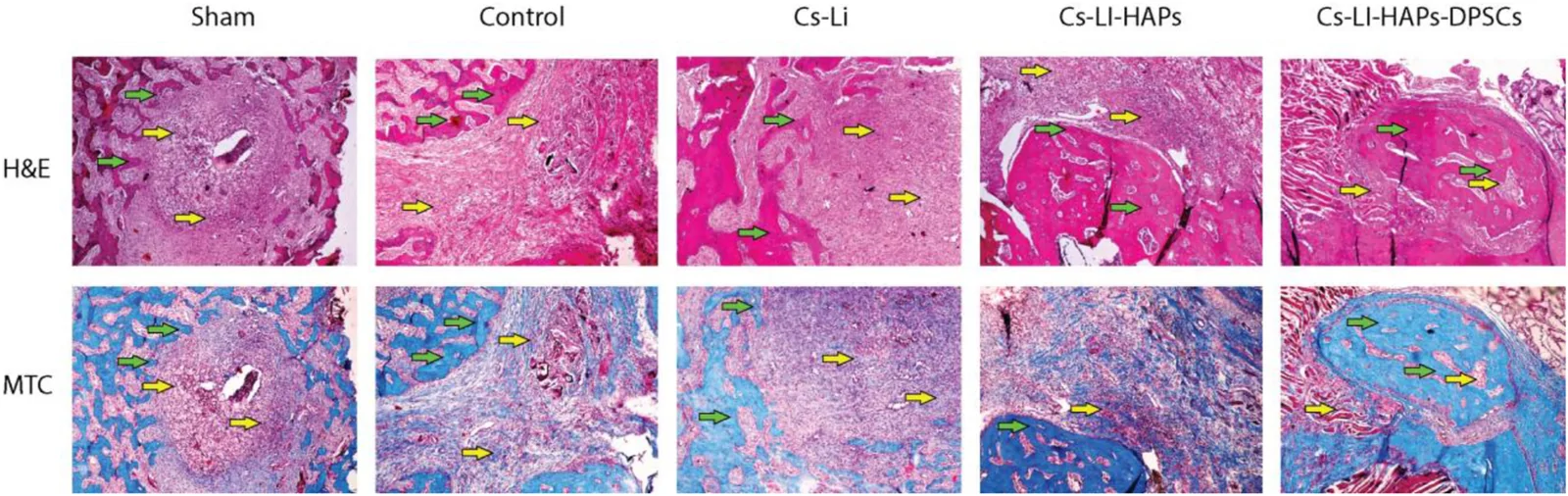
Histopathological analysis of bone regeneration representative H&E (top row) and MTC (bottom row) stained sections of the defect site across experimental groups. Both Sham and Control groups exhibited fibrous connective tissue with minimal, isolated regions of early woven bone. Enhanced regeneration was observed in the Cs-LI and Cs-LI-HAPs groups, characterized by new woven bone formation adjacent to connective tissue, indicating the osteoinductive properties of the herbal extract (LI) and biomimetic HAPs. The most advanced regeneration occurred in the scaffold co-loaded with LI, HAPs, and DPSCs, which featured both woven and mature lamellar bone, an absence of epithelial invasion, and no inflammatory cell infiltration. Green arrows: woven bone; Yellow arrows: connective tissue.

**Fig. 8:**
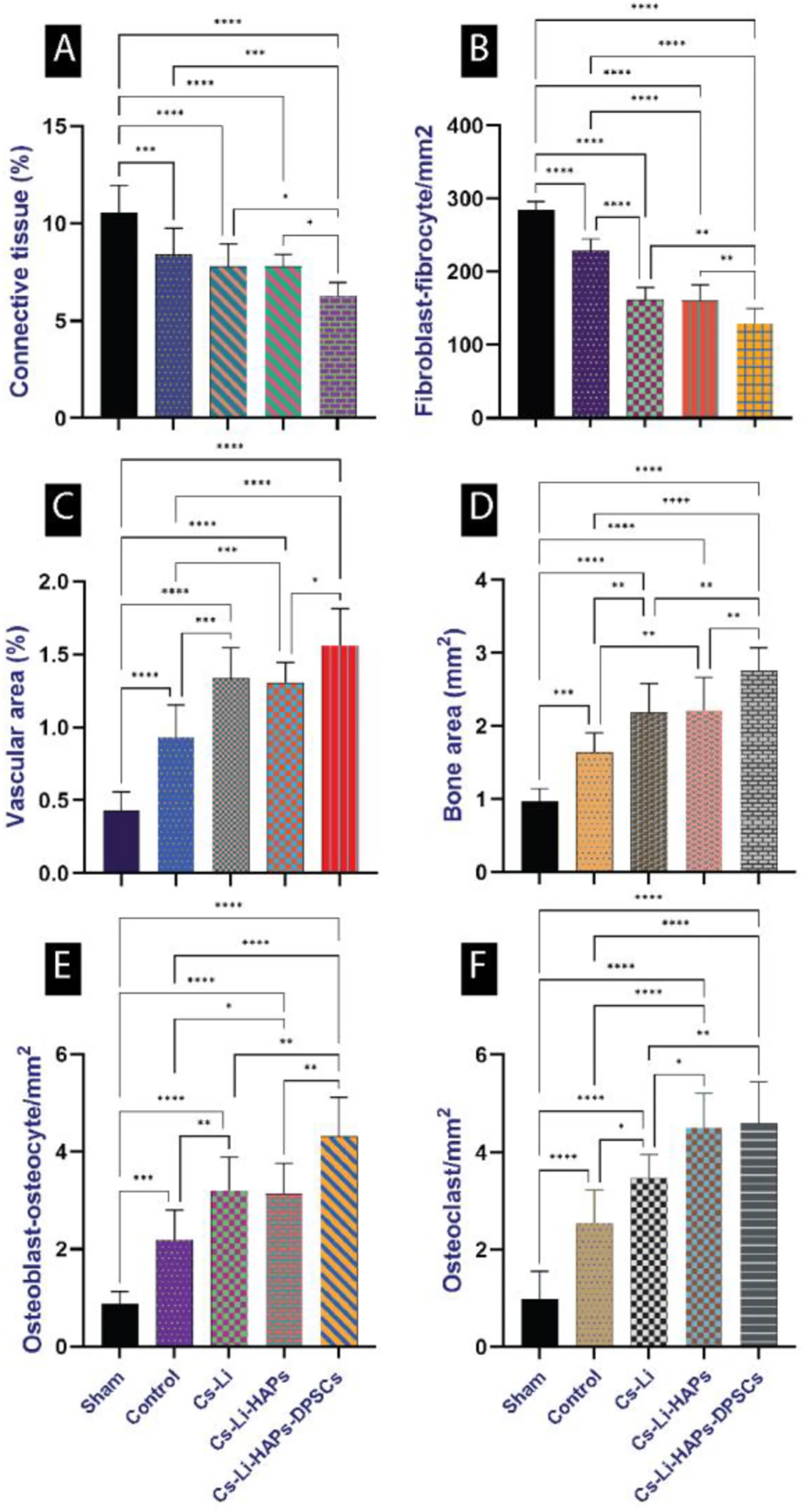
Histomorphometric analysis of bone regeneration parameters. (A) Connective tissue percentage, and (B) fibroblast/fibrocyte density demonstrating decreased indexes in scaffold groups, particularly Cs-LI-HAPs-DPSCs. (C) Vascular area percentage revealing enhanced angiogenesis in scaffold groups, with maximal effect in Cs-LI-HAPs-DPSCs. (D) Bone area percentage, (E) osteoblast/osteocyte, and (F) osteoclast numbers showing significantly improved bone formation and cellular activity in scaffold groups, especially Cs-LI-HAPs-DPSCs. Data demonstrated the synergistic benefits of combined scaffold-cell constructs for bone regeneration. *p < 0.05, **p < 0.01, ***p < 0.001, and ****: p < 0.0001.

Histopathological examination of bone regeneration parameters showed significant differences among the experimental groups (Fig. 7) that demonstrated a significant decrease in connective tissue formation (Fig. 8A) and fibroblast density (Fig. 8B), indicating that inclusion of LI in the nanocomposite also attenuates collagenous responses. Meanwhile, angiogenesis (Fig. 8C), bone area (Fig. 8D), osteoblast (Fig. 8E), and osteoclast (Fig. 8F) activity were increased in the groups containing LI and HAPs. The improved regenerative outcomes observed in this study can be attributed to a combination of factors, each contributing in a distinct manner. The porous three-dimensional structure of the chitosan scaffold provided a suitable substrate for cell attachment, proliferation, and tissue ingrowth, while its interconnected porosity facilitated nutrient diffusion and vascular invasion, and the mechanical integrity of the cross-linked scaffold maintained defect space during the early stages of healing N. In addition to the architectural support provided by chitosan, the incorporation of HAPs contributed to the osteoconductive properties of the composite scaffold; its biomimetic apatite chemistry facilitated chemical bonding to surrounding bone, while its nanotopography stimulated osteogenic differentiation of DPSCs, potentially complementing cellular BMP/Wnt pathways to upregulate RUNX2 expression [41,42]. Furthermore, the controlled degradation rate of HAPs-containing scaffolds (65.48 ± 10.35% at 4 weeks) provided sustained mechanical support during bone formation [43].

Beyond the structural and osteoconductive contributions of the scaffold, the inclusion of *Lawsonia inermis* extract added bioactive functionality to the system. LI may have enhanced bone healing through anti-inflammatory, antioxidant, and pro-osteogenic effects [44]. Its bioactive components (lawsone, gallic acid) may have abrogated early inflammatory cascades and spared a profibrotic response to ultimately facilitate DPSC differentiation. Concomitantly, LI antioxidants (e.g., flavonoids) scavenged ROS, thus shielding DPSCs and their osteoprogenitors from oxidative stress, a component essential for MSCs survival at hypoxic fracture sites [9].

Importantly, the most significant bone regeneration was observed in the Cs-LI-HAPs-DPSCs group, highlighting the critical role of DPSCs in enhancing osteogenesis. This was particularly striking in this cohort, as collagen fibers tended to be most attenuated among the samples studied (Fig. 7, Fig. 8A), and vascularization was most developed in this group (Fig. 7, Fig. 8C), which to a certain extent was consistent with the pro-angiogenic effect of DPSCs. These cells secrete vascular endothelial growth factor (VEGF), basic fibroblast growth factor (bFGF), and angiopoietin-1 (Ang-1) stimulating endothelial cell migration and formation of a vascular network, essential for nutrient delivery to the site of repair and attraction/homing of osteoprogenitor cells [41,42]. This angiogenic enhancement was probably also responsible for the group's increased mineralization (Fig. 8D), osteoblast/osteocyte (Fig. 8E), and osteoclast counts (Fig. 8F), as vascularity is required for osteogenesis during both endochondral and intramembranous ossification**.** The strong osteo-inductivity of DPSCs is likely related to their multi-lineage differentiation potential and paracrine functions. DPSCs presumably utilized BMP-2/Wnt/β-catenin pathways to promote expression of RUNX2 and osterix (OSX), leading to osteoblast differentiation while inhibiting SOX9 for chondrogenic lineage and reducing fibrosis [45]. At the same time, their immune-modulatory function through TLR signaling and release of anti-inflammatory cytokines (e.g., CCL5, CXCL9, CXCL10) may have dampened excessive inflammation, ameliorated fibrotic scarring, and established a pro-regenerative milieu [46,47].

Overall, the improved bone regeneration observed in the Cs-LI-HAPs-DPSCs group may result from the combined effects of multiple factors, including the scaffold architecture, HAPs, LI, and DPSCs. The integration of osteoconductive HAPs, bioactive LI, and osteogenic DPSCs within a chitosan matrix provides a favorable microenvironment for bone healing. These proposed mechanisms are consistent with previously reported pathways in the literature [[45], [46], [47]]; however, direct evaluation of these signaling cascades was beyond the scope of the present study and remains an important direction for future investigation. These results suggest the biphasic effects of DPSCs on bone regeneration: they work through (1) direct osteolineage differentiation and (2) angiogenesis and immunomodulating trophicity. Furthermore, the Cs-LI-HAPs scaffold enhanced these effects as it offered a bio-mimicking niche for cell adhesion and signaling.

## 4 Conclusion

For bone engineering applications, this study effectively created and characterized a novel composite scaffold combining Cs, LI extract, HAPs, and DPSCs. The successful synthesis of biomimetic HAPs particles with the right chemical composition was confirmed through comprehensive characterization. Both LI and HAPs significantly improved the structural and biological properties of Cs scaffolds compared to pure Cs scaffolds. The composite scaffold exhibited optimal porosity, enhanced mechanical strength, controlled degradation kinetics, and favorable surface wettability, all essential properties for bone tissue engineering applications. Additionally, the scaffolds seeded with DPSCs demonstrated outstanding hemocompatibility and biocompatibility. Therefore, we conclude that the Cs-LI-HAPs composite scaffold included DPSCs has the biological and physicochemical characteristics required to function as a suitable platform for bone regeneration.

## Ethics declaration

Written informed consent to take part in the study and to publish the article has been obtained from all participants or their legal representatives. The privacy rights of participants have been observed.

This study included organ or tissue donors. This study includes human biological material and consent was obtained by donors, or their next of kin or legal representatives, for use in this study and for publication of the article. The samples used in this research were not sourced from executed prisoners or prisoners of conscience.

This study was performed in compliance with relevant laws, regulatory frameworks and guidelines where the research took place. This study was conducted in accordance with ARRIVE. This study was approved by the institutional ethics committee of Shiraz University of Medical Sciences. (Approval No. IR.SUMS.AEC.1403.051)

## Data availability

Data will be made available on request.

## Ethics approval and consent to participate

All procedures were performed in accordance with Shiraz University of Medical Sciences guidelines and regulations. All experiments were approved by the ethical committee of Shiraz University of Medical Sciences (Approval No. IR.SUMS.AEC.1403.051).

## Authors' contributions

**Maryam Karandish:** Supervision, Project administration, Funding acquisition, Writing - Review & Editing; **Golnaz Ziaei:** Methodology, Writing - Review & Editing; **Fatemeh Akbarizadeh:** Methodology; Negar Azarpira: Methodology; **Shahrokh Zare:** Methodology; **Ahmad Vaez** and **Nader Tanideh:** Supervision, Conceptualization, Data Curation, Methodology, Writing - Original Draft, Writing - Review & Editing. All authors reviewed the manuscript.

## Declaration of generative AI and AI-assisted technologies in the writing process

During the preparation of this work the authors used deepseek in order to improve readability and language editing. After using this tool, the authors reviewed and edited the content as needed and take full responsibility for the content of the published article.

## Funding

Vice-Chancellory of Research 10.13039/501100004320Shiraz University of Medical Sciences supported this research (Grant#30184).

## Declaration of competing interest

The authors declare the following financial interests/personal relationships which may be considered as potential competing interests: Ahmad Vaez reports financial support was provided by Shiraz University of Medical Sciences. Ahmad Vaez reports a relationship with Shiraz University of Medical Sciences that includes: employment. If there are other authors, they declare that they have no known competing financial interests or personal relationships that could have appeared to influence the work reported in this paper.
